# Binding kinetics of ligands acting at GPCRs

**DOI:** 10.1016/j.mce.2019.01.018

**Published:** 2019-04-05

**Authors:** David A. Sykes, Leigh A. Stoddart, Laura E. Kilpatrick, Stephen J. Hill

**Affiliations:** a Cell Signalling and Pharmacology Research Group, Division of Physiology, Pharmacology and Neuroscience, School of Life Sciences, University of Nottingham, Nottingham, UK; b Centre of Membrane Proteins and Receptors (COMPARE), University of Birmingham and University of Nottingham, Midlands, UK

**Keywords:** GPCR, Binding kinetics, Association, Dissociation, 5HT2B, serotonin receptor 2B, β_2_AR, β_2_ adrenoceptor, BRET, bioluminescence resonance energy transfer, GPCR, G protein-coupled receptor, HAC, heavy atom count, *K*_*d*_, equilibrium dissociation constant, *k*_*f*_, forward rate coefficient, *k*_*off*_, dissociation rate constant, *k*_*on*_, association rate constant, *k*_*on(obs)*_, observed rate of drug association, *k*_*r*_, reverse rate coefficient, PD, pharmacodynamics, RET, resonance energy transfer, SKR, structure kinetic relationship, TR-FRET, time resolved fluorescence resonance energy transfer

## Abstract

The influence of drug-receptor binding kinetics has often been overlooked during the development of new therapeutics that target G protein-coupled receptors (GPCRs). Over the last decade there has been a growing understanding that an in-depth knowledge of binding kinetics at GPCRs is required to successfully target this class of proteins. Ligand binding to a GPCR is often not a simple single step process with ligand freely diffusing in solution. This review will discuss the experiments and equations that are commonly used to measure binding kinetics and how factors such as allosteric regulation, rebinding and ligand interaction with the plasma membrane may influence these measurements. We will then consider the molecular characteristics of a ligand and if these can be linked to association and dissociation rates.

## Highlights

• Ligand binding kinetics can be measured using a variety of methods.
• Kinetics can be influenced by the local environment surrounding the receptor.
• The molecular determinants of kinetics rates are beginning to be understood.
• Both *k*_*on*_ and *k*_*off*_ should be considered during drug discovery.

## 1 Introduction

The phrase ‘Corpora non agunt nisi fixata’ or ‘a drug will only act when bound to its target’ is perhaps the most well-known and influential phrase within the field of drug-receptor pharmacology and was coined by the renowned German pharmacologist Dr Paul Ehrlich. Knowing the life-time of the interaction of a drug with its target receptor through measuring its kinetic parameters, is therefore crucial to understanding the full pharmacological effect of a drug and to progress knowledge of its mode of action. A better understanding of the effect of kinetic parameters on drug action is slowly emerging and suggests that the association rate of a ligand with its receptor (*k*_on_) may be just as important as the length of time that the ligand is bound (residence time (1/*k*_off_)) in dictating the action of drugs *in vivo*.

One of the most successfully targeted class of receptors for the development of pharmaceuticals are the cell surface G protein-coupled receptors (GPCRs) with over 26% of currently approved pharmaceuticals having a GPCR as its main physiological target (Garland, 2013). However there is still considerable interest in the development of new compounds that target GPCRs as those currently marketed only target 10% of the GPCR superfamily. Many of these remaining GPCRs have been implicated in a variety of diseases and there have been many attempts to generate compounds that act at these receptors but many have failed in clinical trials often due to a lack of efficacy (Kola and Landis, 2004; Waring et al., 2015). The influence of drug-receptor binding kinetics has often been overlooked during the development of new therapeutics but there is a growing understanding that an in-depth knowledge of binding kinetics at GPCRs is required to successfully target this class of proteins (Copeland, 2016; Schuetz et al., 2017). This review will discuss the experiments and equations that are commonly used to measure binding kinetics and the factors that may influence these measurements. We will then consider the molecular characteristics of a ligand and if these can be linked to association and dissociation rates.

## 2 Equilibrium affinity, association and dissociation rates

**Fig. 1:**
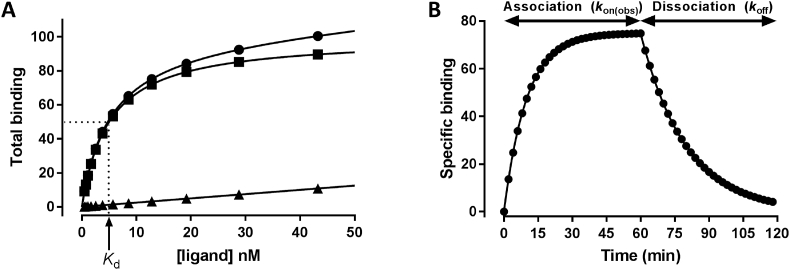
**Simulated saturation and kinetic binding curves.** Saturation and kinetic binding curves were simulated in GraphPad prism assuming R + L ⇋ RL using the one site: total and non-specific binding equation (A) or association then dissociation equation (B) for a ligand with a  *K*_d_ of 5nM (A) or 50nM (B). For (A), the specific binding (squares) B_max_ was set to 100 and *K*_d_ to 5 nM and the non-specific binding (triangles) set to a slope of 0.25. For (B) the *k*_on_ was set to 1 × 10^6^ M^−1^ min^−1^ and *k*_off_ to 0.05 min^−1^, the concentration of ligand to 50 nM and the B_MAX_ to 150, which results in a *K*_d_ of 50 nM. The association phase of the kinetic binding curve is termed *k*_on(obs)_ and is defined as *k*_on_ = (*k*_on(obs)_-*k*_off_)/[ligand].

The equilibrium dissociation constant *(K*_d_*)* is an important pharmacological parameter which describes the concentration of a drug required to occupy 50% of its target receptors at equilibrium. This value was traditionally considered fundamental to understanding structure/function relationships which in turn has enabled more efficient drug design. *K*_d_ is often determined directly from saturation type ligand-binding experiments, where increasing amounts of a labelled ligand are added and the levels of bound ligand are measured (Fig. 1). From these experiments, *K*_d_ is defined as the concentration of ligand producing half maximal specific binding (Fig. 1), once non-specific binding has been taken into account. *K*_d_ by definition is an equilibrium parameter and when measured in a closed system, such as is the case for simple binding reactions, the concentration of reactants reaches the point that the forward and reverse reactions are in balance. This means that *K*_d_ is only half the story as it is a composite of two kinetic parameters, the association rate constant (*k*_on_) and dissociation rate constant (*k*_off_) of a ligand and is therefore defined as a ratio of these rate constants (*K*_d_ = *k*_off_/*k*_on,_ Fig. 1).

The dissociation rate constant, *k*_off_, describes the dissociation of a single species and is therefore a unimolecular or first order rate constant and describes the rate of drug-target dissociation (Fig. 1). *k*_off_ is independent of the local concentration of free drug and is entirely dependent on specific interactions between the drug and its target which in the case of GPCRs is often a binding pocket. As *k*_off_ is independent of ligand concentration it is expressed in units of s^−1^ (or min^−1^).

The association rate constant, *k*_on_ is a bimolecular or second order rate constant as it describes the rate at which two molecules (the drug and the receptor) bind to each other, estimating the rate of drug-target complex formation. Binding reactions are said to be either diffusion limited, which describes a process so fast that the reactants need only collide with one another for binding to occur, or encounter limited indicating that before a reaction can occur the reactants must undergo some degree of reorganisation, which may be reorientation and/or desolvation. As a consequence *k*_on_ cannot be greater than the diffusion limit of 1 × 10^9^ M^−1^ s^−1^ which represents the maximum rate at which two molecules can move through an aqueous environment and eventually collide (Alberty and Hammes, 1958). When *k*_on_ approaches the diffusion limit essentially all encounters of unbound receptor and molecule involved result in successful binding.

**Fig. 2:**
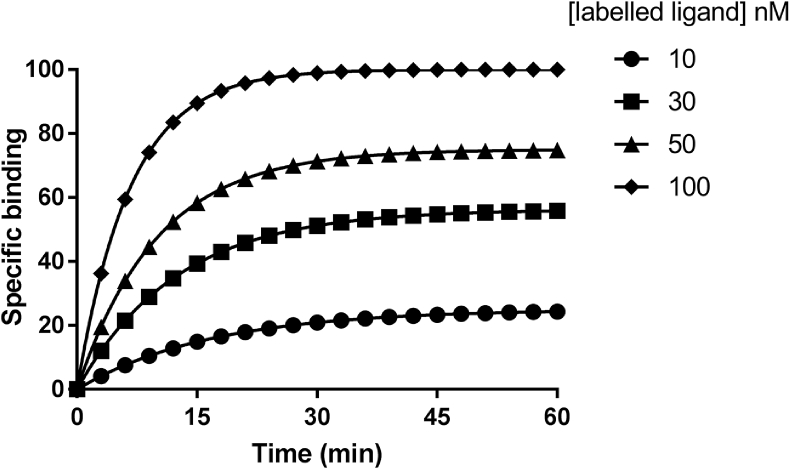
**Simulated association kinetics binding curves.** Typical association kinetic binding curves were generated in GraphPad Prism assuming R + L ⇋ RL. The equation ‘two or more concentrations of hot ligand’ was used with tracer concentrations fixed at 10, 30, 50 and 100 nM. The *k*_off_ value was set to 0.05 min^−1^ and *k*_on_ to 1 × 10^6^ M^−1^ min^−1^ and B_max_ set to 150. This is an example of the data obtained in association kinetic experiments with a ligand with a *K*_d_ of 50 nM.

As shown in Fig. 1, the measured association phase is termed *k*_on(obs)_ rather than *k*_on._ *k*_on(obs)_ is the observed rate of drug association and is composite of both association and dissociation rates. In addition, *k*_on(obs)_ is highly dependent upon drug concentration; a higher concentration of ligand will result in a faster *k*_on(obs)_ (Fig. 2). Therefore, *k*_on_ is equal to (*k*_*on(*obs)_-*k*_off_)/[ligand] and is expressed as M^−1^ s^−1^ (or M^−1^ min^−1^).

## 3 How are kinetics measured at a GPCR?

### 3.1 Radioligand binding assays

Traditionally the kinetics of drugs have been studied directly using radiolabelled forms of the compounds (Insel and Stoolman, 1978; Bürgisser et al., 1981; García-Sevilla et al., 1981). In such experiments, membranes containing the receptor of interest are incubated with increasing concentrations of radioligand in the presence and absence of an excess of competitor used to define non-specific binding and the levels of binding monitored at different time points (Fig. 2). The resulting data can be fitted to equations to derive the *k*_on_ and *k*_off_ rate constants of the radioligand. The dissociation rate can be monitored by pre-incubating the receptors with radioligand, with the dissociation phase being initiated by adding an excess of unlabelled competitor or through the process of ‘infinite’ tracer dilution (Fig. 1). These experiments are likely to produce the most direct information about a drug's association and dissociation rates however they are not without their problems especially when we consider that some drugs based on endogenous ligands such as monoamines have low affinities for their orthosteric binding site relative to their affinities for non-specific sites that can result in a small window of specific binding (Auberson et al., 2016; Laruelle et al., 2003). Dissociation experiments based on addition of a high concentration of a competing ligand also assume that there is no allosteric component to the ligand-receptor binding interactions. This has been demonstrated for receptors such as the chemokine receptors, CCR2 and CCR5, and the adenosine A_3_ receptor, whereby the presence of an unlabelled ligand changes the dissociation constants of labelled ligands under infinite dilution conditions (Springael et al., 2006).

Another problem with the above described procedures is that every compound of interest must be custom synthesised and labelled with a radioisotope which is cost and time prohibitive, and therefore restricts the number of compounds which can be readily tested. An alternative method is to use a single radioligand with high affinity and selectivity for the receptor and utilise the equations provided by Motulsky and Mahan allowing the kinetics of an unlabelled ligand in competition with the radioligand to be determined (Motulsky and Mahan, 1984, Fig. 3). This method has been applied successfully by ourselves and others to determine the kinetics of both antagonists (Dowling and Charlton, 2006; Stoddart et al., 2018, Sykes et al., 2014a) and agonists (Guo et al., 2012; Sykes et al., 2009; Sykes et al., 2012a) acting at a variety of GPCRs and more recently at non-GPCR targets (Yu et al., 2015). Alternatively, multistep washout experiments (see Vauquelin and Van Liefde., 2012 for a comprehensive review) have been employed to compare the dissociation rates of unlabelled antipsychotics (Leysen and Gommeren, 1984; Tresadern et al., 2011) and mu opoid receptor specific ligands (Ilien et al., 1988) although this method is qualitative and only allows relative differences in dissociation rates to be approximated.

**Fig. 3:**
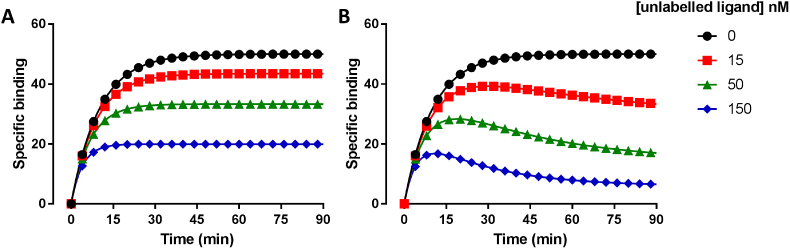
**Simulated competition association binding curves fitted using the Motulsky and Mahan equation to determine the kinetics of unlabelled ligands.** Typical kinetic tracer profile observed with an unlabelled ligand with similar kinetics to the labelled ligand **A** or with a slower *k*_off_ in comparison to the labelled ligand **B.** Simulations were generated in GraphPad Prism using the Motulsky and Mahan equation. For both **A** and **B** the concentration of the labelled ligand (L) was set to 50 nM, *k*_on_ (K1) to 1 × 10^6^ M^−1^ min^−1^, *k*_off_ (K2) to 0.05 min^−1^ and the concentration of the unlabelled ligand (I) set to 15 nM (red squares), 50 nM (green triangles) and 150 nM (blue diamonds). For **A**, the *k*_on_ (K3) was set to 1 × 10^6^ M^−1^ min^−1^ and *k*_off_ (K4) to 0.05 min^−1^. For **B** the *k*_on_ (K3) was set to 1 × 10^6^ M^−1^ min^−1^ and *k*_off_ (K4) to 0.01 min^−1^ thus demonstrating the classic tracer ‘overshoot’, observed when an unlabelled ligand has a slower *k*_off_ than the labelled ligand.

When used with high affinity ligands, these type of experiments provide a high degree of sensitivity, nonetheless the use of radioactive ligands as tracers presents a number of challenges. Most compelling is that they cannot be read continuously; for every time point required to generate kinetic data a separate experimental condition needs to be prepared. In addition, classic radioactive binding assays cannot be performed in a homogeneous format as the bound fraction of radioligand needs to be separated from the free fraction which is usually achieved by filtration to trap the membranes onto filter paper or by multiple wash steps when using cells grown in mono-layer. Another major drawback with radioactive probes is their hazardous nature which can be restrictive in terms of safe exposure levels, and impose expensive radioactive waste disposal procedures and delimitation of working areas. This makes these assays more difficult to perform in a high throughput screening mode and therefore less attractive to implement in a drug discovery setting. Recently, bead-based scintillation proximity assays have been developed specifically to measure binding kinetics (Xia et al., 2016) under homogeneous conditions. However, this does not overcome the safety issues associated with the use of radioligands. In addition, such assays suffer from bead settling which can lead to inaccurate estimates of binding at the very early time points with these time points being often critical for accurate estimations of unlabelled compound kinetic parameters.

### 3.2 Fluorescence based methods

The emergence of fluorescence-based methods offers an alternative to radioactive binding assays and represents a potentially higher-throughput approach to assess unlabelled ligand kinetics. Inherently, the fluorescent probes themselves are safer making assays easier to implement plus the costs associated with waste disposal compared to radiometric assays is minimal. High affinity fluorescent tracers have now been designed and synthesized for numerous receptors and are now readily available from an ever increasing number of commercial suppliers (Ciruela et al., 2014; Vernall et al., 2014). It is important to note that the addition of a fluorophore to a ligand will increase its molecular weight and can change its physicochemical properties (e.g. hydrophobicity). This may increase steric hindrance resulting in significantly altered pharmacological properties (Vernall et al., 2014). However, even a modest reduction in affinity need not reduce the effectiveness of the ligand in pharmacological binding studies provided it remains specific for the intended target protein. Such reductions in affinity may actually provide a more viable tracer for high throughput screening kinetic studies (which ultimately rely on miniaturisation), since they are less prone to tracer depletion, a potential problem in the smaller assay volumes routinely employed (Carter et al., 2007).

**Fig. 4:**
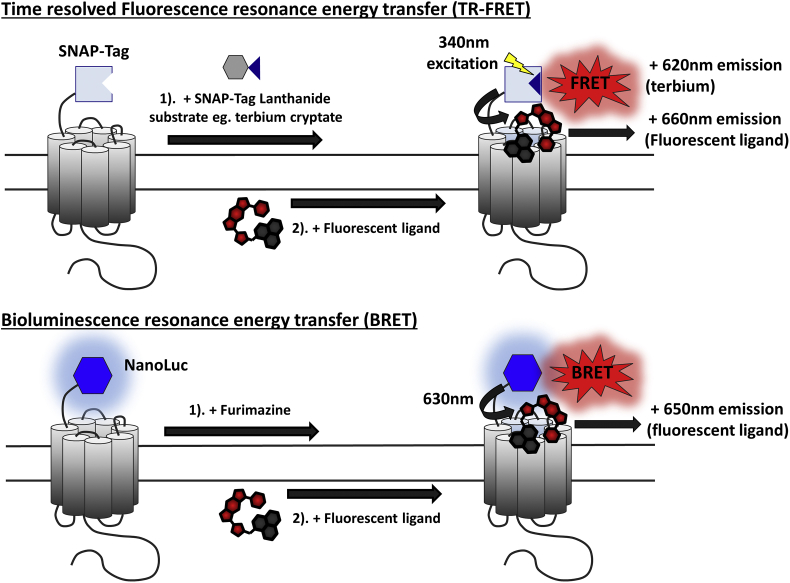
**Schematic illustrating the principles of bioluminescence resonance energy transfer (BRET) and time resolved fluorescence resonance energy transfer (FRET) to measure ligand binding kinetics at G protein-coupled receptors (GPCRs)**. In time resolved fluorescence resonance energy transfer (TR-FRET) assays, lanthanides are used as fluorescent donors as their long emission lifetimes allow measurements to be made following a time delay. This results in increased sensitivity as short-lived background fluorescence is decreased allowing TR-FRET to be used *in situ*. Here, a SNAP-Tag has been engineered onto the N terminus of a GPCR of interest, and the lanthanide donor (illustrated here using terbium cryptate) is delivered to the receptor via a membrane impermeant lanthanide labelled SNAP-Tag substrate (1). The lanthanide label is then excited using laser excitation (eg. ∼340 nm; yellow lightning arrow). If the lanthanide and fluorescent ligand are within close proximity (typically <10 nm), the energy emitted following lanthanide excitation can be transferred to excite the fluorophore of the fluorescent ligand (FRET). Fluorescence emissions from the donor and acceptor can be recorded and used to calculate subsequent FRET ratios. For bioluminescence resonance energy transfer (BRET) assays, a membrane bound receptor of interest (eg. a G protein-coupled receptor (GPCR)), is tagged at its N terminus with a bioluminescent protein donor (eg. NanoLuc). The NanoLuc substrate furimazine is then added for 10–15min and basal BRET measurements are recorded (1). The fluorescent ligand of interest (acceptor) is then added (2). If the fluorescent acceptor and donor luciferase are in sufficiently close proximity (typically <10 nm) then the energy emitted upon furimazine oxidation (in the form of photons) can be transferred to excite the fluorophore of the fluorescent ligand (BRET). Luminescence and subsequent fluorescence emissions can be recorded and used to calculate BRET ratios.

Direct measurement of the binding of fluorescent ligands to a receptor is possible using confocal microscopy and this has been successfully applied to measure the kinetics of ligands binding to the adenosine A_1_ and A_3_ (May et al., 2010) and the β_1_-adrenoceptor (Gherbi et al., 2015). These techniques are very sensitive and provide a high degree of resolution but are time consuming. Higher throughput methods have been developed to measure levels of fluorescent ligand in a plate reader-based format which are well suited to measure equilibrium binding (Arruda et al., 2017) but the signal-to-noise ratio is often too small to accurately determine binding kinetics due to high non-specific binding of the ligands (Stoddart et al., 2015a). This lack of resolution can be overcome through the use of resonance energy transfer (RET) based methods as a signal is only observed when the fluorescent ligand and energy source (fluorescent or bioluminescent protein) are in close proximity (<10 nm; Fig. 4). Both time-resolved fluorescence- (TR-FRET) and bioluminescence- (BRET) resonance energy transfer techniques have been applied to successfully monitor the real time kinetics of ligand binding to GPCRs (Schiele et al., 2015; Stoddart et al., 2018).

The TR-FRET method utilizes self labelling proteins, such as SNAP, CLIP, ACP (all New England Biolabs respectively) or Halo (Promega Corporation)-tagged receptors which are then labelled with a substrate carrying a lanthanide cryptate such as terbium (Cisbio Bioassays) which forms a covalent bond. FRET detection then occurs following the transfer of energy from the terbium donor, to an acceptor fluorophore attached to a ligand selective for the receptor of interest (Schiele et al., 2015). The use of elements such as terbium which have long-lived fluorescence, allows for a time delay (50–150 μs) between excitation and measurement of the resulting fluorescence from the donor and acceptor. This reduces auto-fluorescence from other components within the sample and improves signal to noise ratios. In the BRET methodology, the receptor is tagged with the small, bright luciferase NanoLuc which in the presence of its substrate furimazine produces bioluminescent light (Hall et al., 2012). Then if in close enough proximity (<10 nm), the resonance energy from the NanoLuc can be transferred to a fluorescent ligand bound to the receptor. The overall approach of both energy transfer based techniques is similar to the classic competitive association radioligand binding methodology described above, except that a modified Motulsky and Mahan competitive binding model is employed to take into account apparent bleaching of the fluorophore in the TR-FRET method caused by laser excitation (Schiele et al., 2015).

The main advantage of RET techniques is that the signal recorded is dependent on very close proximity between the two labelled species and as a consequence is specific for the binding event between the labelled ligand and the tagged protein of interest. This means that separation of bound and unbound label is no longer necessary and thus this homogeneous assay format enables higher throughput (Emami-Nemini et al., 2013). Due to the plate reader based format of RET assays, they can also achieve a greater kinetic resolution than radioligand based techniques as reads can be made every 5 s if required. Both BRET and TR-FRET techniques can be used with a range of fluorophores making them compatible with the majority of fluorescent ligands available for GPCRs. However an inherent limitation of RET techniques is the need to label the receptor of interest using a exogenous substrate, which potentially risks altering the receptor when compared to its wildtype counterpart and can be a time consuming process. Nevertheless, RET techniques have been successfully applied to a variety of GPCRs (Klein Herenbrink et al., 2016; Nederpelt et al., 2016; Stoddart et al., 2015b) and to the receptor tyrosine kinase, vascular endothelial growth factor receptor 2 (VEGFR2; Kilpatrick et al., 2017; Peach et al., 2018) and have made the determinination of kinetics more attainable for many researchers working with membrane bound receptors.

## 4 Factors that can influence binding kinetics

**Fig. 5:**
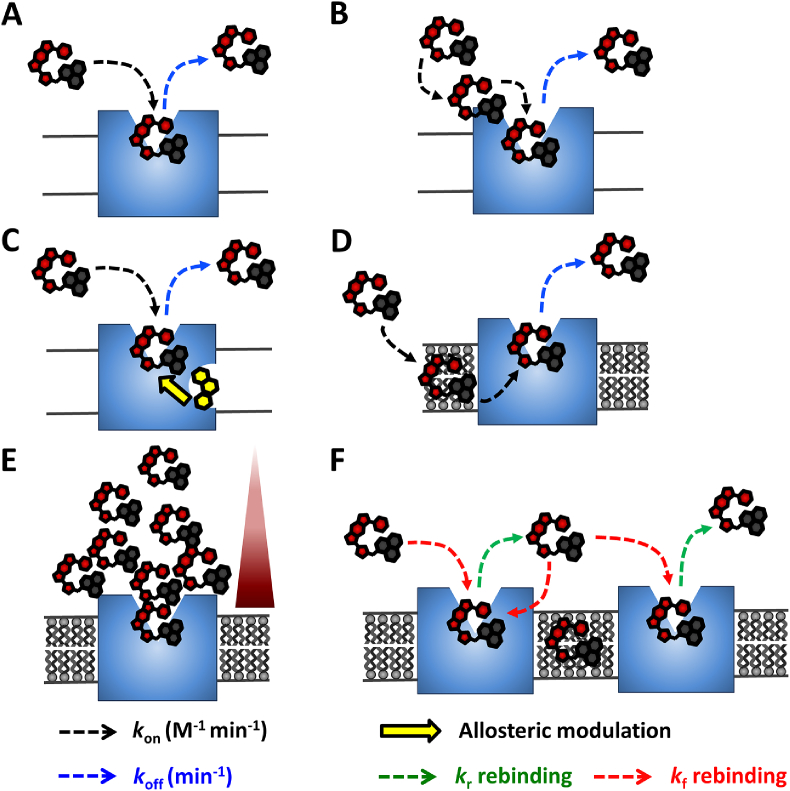
**Schematic illustrating how ligand binding kinetic association and dissociation rates can be influenced by receptor and cellular factors.** The kinetics of ligand binding to its cognate receptor can be defined in respect to its association rate (termed the *k*_on_ rate for that respective ligand; dashed black arrows) and its dissociation rate into aqueous solution (termed the *k*_off_ rate; dashed blue arrows). Ligand binding can be a single step process (**A**), or involve multiple steps whereby a ligand must initially interact with a remote site on the receptor prior to its slower access into the deeper orthosteric binding site (**B**). Many membrane bound receptors, such as GPCRs, contain additional allosteric sites which are topographically distinct from that of the orthosteric ligand binding site **(C)**. If a secondary ligand is bound to an allosteric site (ligand shown in yellow), it has the potential to substantially modulate (increase or decrease) the association and/or dissociation rates of the tracer ligand at the orthosteric site (yellow arrow). Ligands with high lipophilicity are often reported to interact with the lipid bilayer in order to access the orthosteric ligand binding site **(D)**. Due to the potential requirement for lateral diffusion through the bilayer, the association rates measured in kinetic assays may not wholly represent the individual microkinetic events that govern binding of these ligands. Local ligand concentrations above cells have been shown to be affected by receptor concentration and cellular factors such as interaction with the lipid bilayer and extracellular matrix components such as heparan sulfate proteoglycans **(E)**. Ligand concentrations should therefore not be assumed to be freely diffusing uniformly in solution, but may instead be concentrated as gradients (red triangle) around receptors with the potential to substantially alter association rates. A further phenomenon that may affect measured ligand association and dissociation rates is drug-receptor rebinding **(F)**. Here a reversibly bound ligand dissociates according to the ‘effective’ reverse rate coefficient ((*k*_r_) dashed green arrows)) from its cognate receptor but is then able to rebind to the same or a nearby receptor according to the ‘effective’ forward rate coefficient ((*k*_f_) dashed red arrows) before diffusing away into bulk aqueous solution. The rebinding process is favored by ligands with ‘fast’ association kinetics and is influenced by the diffusion rate of the ligand, receptor expression levels and localised restrictions on free ligand diffusion (eg. Local receptor compartment geometry, with contributions from the lipid bilayer and extracellular matrix components).

In addition to a direct molecular interaction of a ligand with the orthosteric binding site of a receptor, there are many factors that can influence the kinetics of ligand binding. As with any mathematical equation, those used to analyse data obtained in kinetic experiments require certain assumptions to be met in order that the models applied are valid. In the following section, we will discuss the factors that can change kinetic rates and how this may influence the assumptions we make when fitting association and competition association experiments (Fig. 5). The following examples are not exhaustive and there are other factors such as ligand depletion (Carter et al., 2007; Hulme and Trevethick, 2010), physiologically relevant ions (Katritch et al., 2014) and direct effects of membrane lipids (Dawaliby et al., 2016; Bruzzese et al., 2018) which are known to affect equilibrium binding parameters but to date their influence on kinetic parameters have not been studied.

### 4.1 Single step binding

The process of ligand binding as described by the equations used to formulate competition association binding models assumes that ligand association and dissociation occurs in a single step (Fig. 5A, Motulsky and Mahan, 1984). However molecular dynamic simulations, spectroscopy and crystallography all strongly suggest the presence of metastable and/or intermediate states that occur during a multi step ligand-GPCR binding process (Fig. 5B, Latorraca et al., 2017). The presence of such states along the binding pathway are likely to signal what is referred to as a reduction in dimensionality, with the ligands pathway trajectory into the binding pocket no longer controlled by the laws of free diffusion (Axelrod and Wang, 1994). The consequence of this will be a change in the ligand's overall measured association rate. These metastable and/or intermediate states are likely to be the basis for on-rate mediated subtype receptor selectivity. In such cases, measured on (and off-rates) are simply the net effect of transitions between multiple conformational minima, and require a more sophisticated treatment than a simple two state model (Tang et al., 2017). Similarly off-rate mediated selectivity has been attributed to Coulombic repulsion, which occurs when two molecules of the same charge come into close proximity. The slow off rate of tiotropium from the M_3_ receptor has been suggested to be due to the presence of a positively charged residue (K523^7.32^) above the exit channel which is thought to mediate Coulombic repulsion between the positively charged ligand and this residue which subsequently slows the exit of the ligand from the receptor. The absence of this charged residue from the closely related M_2_ receptor potentially contributes to tiotropium's faster association and dissociation rates compared to the M_3_ receptor. Ultimately this feature may explain why tiotropium shows comparable affinities for both muscarinic receptor subtypes but exhibits kinetic selectivity for the M_3_ receptor (Tautermann et al., 2013,Sykes et al., 2012b).

### 4.2 Allosteric modulation

Allosteric regulators are molecules which bind to a site distinct to that of the endogenous or orthosteric ligand and by definition alter the binding kinetics of the orthosteric ligand (Christopoulos and Kenakin, 2002. Fig. 5C). Allosteric modulators can have varied effects on kinetic parameters and can change association rates, dissociation rates or both to the same or different degrees (Kostenis et al., 1996; Molderings et al., 2000; Gao et al., 2001). Indeed, numerous examples of endogenous substances acting as allosteric modulators of GPCRs have been described (van der Westhuizen et al., 2015). Another important hallmark of allosteric modulators is that they are probe dependent, which means that they have varying effects depending on the orthosteric ligand used. As with measuring the kinetics of orthosteric ligands, this means that only the effects on a labelled molecule can be measured. To address this, Guo et al. extended the use of the competition association assay to investigate the effect of allosteric modulators at the adenosine A_1_R (Guo et al., 2014). In this study, the radiolabelled antagonist ([^3^H]DPCPX) was used to measure the kinetics of unlabelled orthosteric ligands in the presence and absence of two different allosteric modulators. As the equations used to analyse data obtained in competition association assays requires the *k*_on_ and *k*_off_ of the labelled ligand to be fixed, the authors directly measured the kinetics of [^3^H]DPCPX in the presence of the two modulators and used these values in subsequent analysis. Importantly they found that there was not a global change in the kinetic constants of the labelled ligand ([^3^H]DPCPX) in the presence of the probes therefore the effect of the modulators on the residence time of other orthosteric compounds could be tested. It was found that the modulators increased the residence time of the agonists CCPA and NECA which demonstrated both the probe dependence of the modulators and that subtle differences could be measured using this methodology. The competition association assay has also recently been used to directly measure the kinetics of allosteric modulators at the metabolic glutamate receptor 2 (Doornbos et al 2017, 2018). As mentioned above as one of the main characteristics of allosteric modulators is that they change either the association or dissociation rates of an orthosteric ligand applying the Motulsky Mahan equation to data obtained using an allosteric modulator needs to be done with caution.

Allosteric modulation of a GPCR does not necessarily need to be by a small molecule, it can also be through interaction with accessory proteins such as G protein and β-arrestins or through dimerization with another GPCR. This has been demonstrated to be the case for the adenosine A_3_ receptor and the β_1_ adrenoceptor (May et al., 2011; Gherbi et al., 2015). For the adenosine A_3_ receptor it was shown that orthosteric ligands enhanced the dissociation rate of a labelled agonist. Through the use of a non-binding mutant of the receptor, it was shown that this effect was due to allosteric modulation across a homodimeric interface (May et al., 2011).

### 4.3 Local ligand concentration and ligand interaction with plasma membrane

One of the main assumptions made in the equations used to analyse kinetic binding experiments is that the ligands are freely diffusing in solution (Fig. 5E). A recent study by Gherbi et al. shed light on the phenomenon of local ligand concentration (Gherbi et al., 2018). Using a fluorescent ligand for the β_2_-adrenoceptor (β_2_-AR), BY-propranolol, in combination with the sensitive microscopy technique fluorescence correlation spectroscopy (Briddon et al., 2017) they were able to quantify the local concentration of fluorescent ligand at different distances from the plasma membrane. They found that the concentration of BY-propranolol increased the closer to the membrane the measurements were made. Crucially they found that this was dependent on the expression of the β_2_-AR, with double the concentration observed in β_2_-AR expressing cells compared to non-transfected cells. The equations used to calculate affinity from both equilibrium and kinetic experiments requires knowledge of the free concentration of the labelled ligand used. The analysis also assumes that the concentrations of both labelled and competing ligand are constant and not changed by ligand-binding to the receptor or proximity to the receptor. Consequently if the local concentration is higher than the bulk added concentration then this will lead to an apparent overestimation of affinity. For the β_2_-AR, using the concentration determined closest to the membrane the affinity of BY-propranolol was 25-fold lower than that calculated with the bulk added concentration (Gherbi et al., 2018). Although technically challenging, this study highlights the need to consider the influence of the local concentration of ligand when determining binding kinetics and that receptors expressed in different cells and at different expression levels may also affect the concentration of a specific ligand.

In addition to non-uniform distribution of ligands in aqueous solution, ligands can interact directly with the lipids in the plasma membrane thus altering their diffusion characteristics and potentially concentrating them around a particular receptor (Vauquelin and Packeu, 2009, Fig. 5D). This has been studied extensively for the β_2_AR to understand a question which has perplexed pharmacologists for the past 20 year or so as to why certain agonists for this receptor are so long acting (Anderson et al., 1994; Sykes and Charlton, 2012). Calculating lipophilicity (logP) from the structure of a molecule is the easiest way to estimate the propensity of a molecule to interact with the lipophilic cell membrane. It has been demonstrated for a variety of ligands that this has particular relevance to receptors where the native ligand is thought to enter the binding pocket via lateral diffusion through the plasma membrane (Fig. 5D). Receptors that have been proposed to utilise this route of entry include the sphingosine-1-phosphate receptor 1 (S1P1, for which a crystal structure is available), opsin and cannabinoid receptors (Hanson et al., 2012; Hildebrand et al., 2009; Hurst et al., 2010). More recently a lipid pathway has been proposed for small molecules binding to the peptide activated receptor PAR1 (Bokoch et al., 2018). Kinetic association parameters for the native ligands have not been measured directly for these receptors and it is entirely possible that the microkinetic parameters may differ from measured parameters due to this process of lateral diffusion.

### 4.4 Rebinding

Drug-receptor rebinding occurs in situations where free diffusion is limited and describes the process whereby a reversibly bound compound rebinds to the same or nearby receptor before diffusing away into bulk aqueous solution (Fig. 5F). Rebinding has been the topic of a number of recent and insightful reviews (Vauquelin 2010, 2016; de Witte et al., 2016, 2017). As such, drugs may be thought to possess an ‘intrinsic on-rate’ which will potentially differ from their ‘measured on-rate’ which is dictated by factors thought to influence the free concentration of a drug immediately surrounding the receptor's orthosteric binding pocket as described in section 5.3 (Sykes et al., 2014a; Gherbi et al., 2018).

Rebinding is characterised by the establishment of a new dynamic equilibrium between the free target and freshly dissociated drug molecules with *k*_on_ and *k*_off_ being replaced by the forward (*k*_f_) and reverse (*k*_r_) rate coefficients of binding (Fig. 5F). Normally when the conditions of free diffusion operate a molecule dissociates according to its dissociation rate constant (or *k*_off_) and it is assumed to simply drift away into bulk aqueous. In contrast when rebinding predominates a drug molecule will undergo multiple encounters with the initial target or nearby receptors in accordance with the estimated effective reverse rate coefficient (*k*_r_) (Vauquelin and Charlton, 2010). *k*_r_ like *k*_off_ is characteristic of a particular ligand but the calculation of *k*_r_ which is situation-dependent takes into account the unique tissue microenvironment of the synapse and integrates both the association and dissociation rate constants to calculate the overall rate of reversal of receptor blockade.

The clinical relevance of this rebinding phenomenon has recently been demonstrated through studying the *in vitro* kinetics of clinically used antipsychotics and relating their measured on-rates to their prolonged receptor occupancy *in vivo* (Sykes et al., 2017; Seeman, 2002). Specifically this study was able to demonstrate that the estimated effective reverse rate coefficient (or *k*_r_) of antipsychotics and not their *k*_off_ better predicts the levels of extrapyramidal side effects observed in patients. Some useful *in vitro* systems have been proposed to gain some understanding of this rebinding phenomenon (Spivak et al., 2006) but ‘micro-anatomical elements’ found only in intact tissue are a prerequisite if the *in vivo* situation is to be truly simulated (Vauquelin, 2010).

Rebinding of ligands is likely to play a role in extending the effects of a drug in situations where high receptor densities are found, and diffusion restriction imposed by the local geometry and composition of the surrounding tissue. The synapse is an extreme example and represents a semi-closed structure which is thought to be responsible for extending the receptor occupancy of antipsychotics, and possibly other centrally acting compounds (Vauquelin, 2010; Sykes et al., 2017).

### 4.5 Intact cell environment

There has been much debate around the use of membranes versus intact or whole cells to study the binding kinetics of drugs (Motulsky et al., 1985, Verheijen et al., 2004; Vauquelin et al., 2015; Sykes and Charlton, 2018). It can be argued that membranes offer the opportunity to study drug-receptor interactions in a single compartment model which is the situation the equations used to determine binding kinetics were originally formulated for. Therefore any such measurements should be free from many of the complications attributed to inaccessible receptor compartments. This situation will be particularly relevant in a competition association binding assay and be potentially exacerbated if the tracer and competitor have vastly different physico-chemical properties. Equally whole cells offer the opportunity to observe physiologically relevant information and may permit a better understanding of the complex drug-target interactions and the potential of complex kinetic binding phenomenon such as ‘rebinding’ to contribute to extended receptor coverage that is not predicted by simple one compartment models (Spivak et al., 2006; Vauquelin et al., 2015).

The ternary complex model for GPCR activation by agonists indicates that the receptor-G protein complex has higher affinity for agonists than receptor alone (De Lean et al., 1980). In the whole cell environment, this agonist-receptor-G protein complex is short lived due to the high intracellular concentration of GTP. Depending on the method used for membrane preparation the concentration of GTP may be low. This would promote a more stable complex of agonist-receptor-G protein as there is minimal GTP present to induce separation of this complex. It is worth noting, however, that homogenates prepared from guinea pig cerebral cortex have been shown to contain vesicles that can maintain a membrane potential (Creveling and McCulloh, 1980; Creveling et al., 1983). This indicates that membrane preparations, unless prepared in hypotonic media in the presence of detergent, will consist of vesicles that could contain high concentrations of GTP. Therefore, it must be emphasised that if agonist-receptor binding kinetics are studied in membranes using the Motulsky-Mahan model then it is important to study these in the presence of GTP or better still stable analogues of GTP such as GTPγS or GppNHp and a detergent to disrupt vesicles. Studies into the kinetic parameters of agonists binding to the muscarinic M_3_ receptor and the adenosine A_1_ receptor have been carried out in the presence of GTP to promote G protein uncoupling (Sykes et al., 2009; Xia et al., 2016). Kinetic parameters of an agonist (NECA) binding to the adenosine A_2A_ receptor were found to be unchanged in the presence or absence of GTP (Guo et al., 2012) whereas for the adenosine A_3_ receptor it was found that the presence of GTP did not change the association rate constant for agonists but did have an effect on the dissociation rate constants (Xia et al., 2018). Determining kinetic rates in the presence and absence of GTP is likely to become increasingly more important as fluorescent tracers with agonist-like properties become more widely available for the study of unlabelled compound kinetics (Klein-Herenbrink et al., 2016; Sykes et al., 2017).

The intact cell environment also maintains the native membrane potential that a GPCR is normally subjected to. A number of GPCRs have been shown to be voltage sensitive and that voltage can alter agonist affinity (Mahaut-Smith and Gurung, 2008; Vickery et al., 2016). One study has demonstrated that voltage can alter the kinetics of muscarinic agonist binding (Ben Chaim et al., 2013). This study required the use of Xenopus oocytes to enable the effect of depolarization to be investigated. Membrane potential is an important physiological control in situations such as at the neuronal synapse and therefore should be a consideration for GPCRs which are expressed in these systems.

## 5 Moving towards understanding receptor kinetics at the molecular level

### 5.1 Drug-receptor dissociation

With the advent of more high-throughput assays and an increased interest in determining binding kinetics at GPCRs, larger data sets at a range of receptors are becoming available. This, coupled with the recent explosion of GPCR structural information, raises the possibility of designing drugs that may drive a clinically favourable kinetic parameter. To do this it is important to understand the factors that may influence specific kinetic properties to more accurately predict structure kinetic relationships (SKR). However, in truth, little is currently known about the structural factors governing the kinetics of molecular recognition.

There are many factors that have been proposed to influence drug-receptor dissocation. It has been suggested that the presence of water shielded hydrogen bonds may be responsible for slow dissociation of certain compounds essentially acting as ‘kinetic traps’ which effectively increase the stability of the drug receptor complex. This effect is proposed to be partly responsible for the slow rate of dissociation of the long acting muscarinic antagonist tiotropium (Tautermann, 2016). This occurs when rupture of shielded hydrogen bonds transpires at slower rates due to these hydrogen bonds being essentially protected from the effects of water (Schmidtke et al., 2011). Similar observations have proposed that the displacement of unfavourable water molecules in the binding pocket may provide a plausible explanation for slow dissociation of certain antagonists from the cannabinoid CB_1_ receptor (Xia et al., 2017). High resolution structural information has led to specific structural features of receptors being suggested to control dissociation of drugs from receptors. For example the residence times of ligands at the adenosine A_2A_ receptor has been shown to correlate with the energy required to break a salt bridge found at the entrance to the A_2A_ receptor orthosteric binding site (Segala et al., 2016). At this receptor, long residence time ligands appear to stabilize the Glu−His ionic interaction, while fast off-rate derivatives were generally predicted to destabilize this salt bridge. Others have suggested that structural movements of both receptor and or ligand may both hinder association rates and concurrently promote slow dissociation rates (Cusack et al., 2015; Buil et al., 2016). In such a scenario the receptor must first change conformation before a particular ligand is able to recognise and bind the receptor. Such a mechanism has been observed with the low affinity slowly associating/dissociating protease activated receptor 2 specific allosteric ligand AZ8838. In this example ligand entry and exit simulations suggest that rotation of the side chain of His227^ECL2^ is required to facilitate ligand entry/release through the orthosteric pocket (Cheng et al., 2017). It is likely that dissociation is a combination of many of the above factors. Further molecular and pharmacological studies will lead to a greater understanding of the dissociation process and how to dial in the required dissociation constant.

**Fig. 6:**
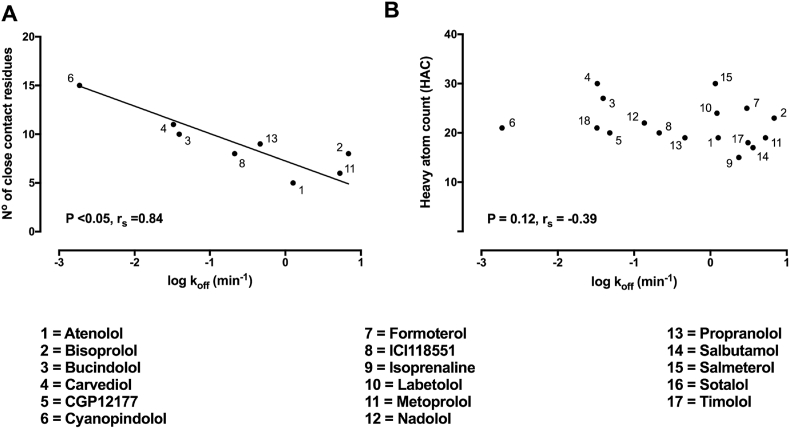
**Correlating β**_**2**_**adrenergic ligand kinetic properties with number of close contact residues and heavy atom count (HAC).** Correlation plot showing the relationship between β_2_ adrenergic compound **(A)** log *k*_off_ and the number of close contact residues in the orthosteric binding pocket, **(B)** log *k*_off_ and the physicochemical property HAC. Kinetic data are presented as mean from three or more experiments. Close contact residue information is taken from Audet and Bouvier (2008) and Warne et al. (2008). All kinetic data used in these plots are detailed in Sykes et al. (2014). Physicochemical properties data was obtained from the National Center for Biotechnology Information. PubChem Compound Database; https://pubchem.ncbi.nlm.nih.gov/compound/. Data was analysed using linear regression in GraphPad Prism v.7 and the relationship between two variables was assessed using a two-tailed Spearman's rank correlation allowing the calculation of the correlation coefficient, r_s_. A P value of 0.05 was used as the cutoff for statistical significance and relationships depicted as trend lines.

Rather than specific interactions that a molecule makes at the molecular level, it may be that more general physicochemical properties of a ligand could go some way to predict kinetic rate constants. Molecular weight, lipophilicity (clogP) and rotational bonds have all been proposed to affect residence time (Miler et al., 2012). As more GPCR structural information becomes available it may become routinely possible to explore the number of close contact residues a particular compound makes within the binding pocket. To illustrate the potential impact of this, we have taken kinetic data for the β_2_AR (Sykes et al., 2014) and correlated *k*_off_ with the number of close contact residues each of these compounds is predicted to make from molecular modelling (Audet and Bouvier, 2008; Warne et al., 2008). The number of close contact residues correlates well with the measured off-rates of eight β_2_AR specific compounds for which close contact information is available (Fig. 6A). Interestingly the heavy atom count (HAC), which is an indicator of increasing molecular weight, was not correlated with *k*_off_ emphasising that residence time (1/*k*_off_) is perhaps more about the quality of interactions made by the ligands in the binding pocket rather than just a general increase in the bulk of a particular molecule (Fig. 6B). With ever increasing numbers of large data sets and high resolution structural information, it is likely that it will become easier to predict kinetics parameters for a given molecule.

### 5.2 Drug-receptor association and its role in pharmacodynamics

The association process has received much less attention than the dissociation binding step as a factor important in the pharmacodynamic (PD) properties of drugs. However evidence is slowly emerging to suggest that the measured association rate may be just as important as the dissociation rate of a drug in determining the overall PD properties of drugs (de Witte et al., 2016). Until recently it was thought that molecules within a chemical series would share very similar association rates and that affinity (or *K*_d_) changes were driven mainly by changes in the magnitude of the dissociation rate (Tummino and Copeland 2008; Núñez S et al., 2012). However, there are many examples where relatively minor changes in structure lead to a significant change in the magnitude of *k*_on_, and therefore directly to a change in affinity (Guo et al., 2012; Sykes et al., 2014). Factors which are thought to affect the association process include diffusion, desolvation and the molecular orientation of the molecule (Copeland et al., 2006; Núñez S et al., 2012; Pan et al., 2013). Theoretically, drugs with non-polar substituents, thus making them more lipophilic, should find it easier to lose and displace water on entry to the binding pocket. This may partly explain the propensity for lipophilic compounds to show increased measured on-rates (Sykes et al., 2014). Such a scenario is also supported by molecular dynamic studies of the β_2_AR which showed that ligand association is affected by the ease with which ligands lose their associated water shells (so called dewetting) and evacuate it from the ligand binding pocket (Dror et al., 2011) with water acting essentially as a barrier to successful ligand binding. This is also supported by studies on heat shock protein (HSP90), where polar substituents were introduced on to molecules to slow binding association (Schuetz et al., 2018). Apart from the introduction of polar groups it may also be possible to introduce groups that create steric clashes within the binding pocket as a mechanism of introducing on-rate driven receptor selectivity (Spagnuolo et al., 2017).

**Fig. 7:**
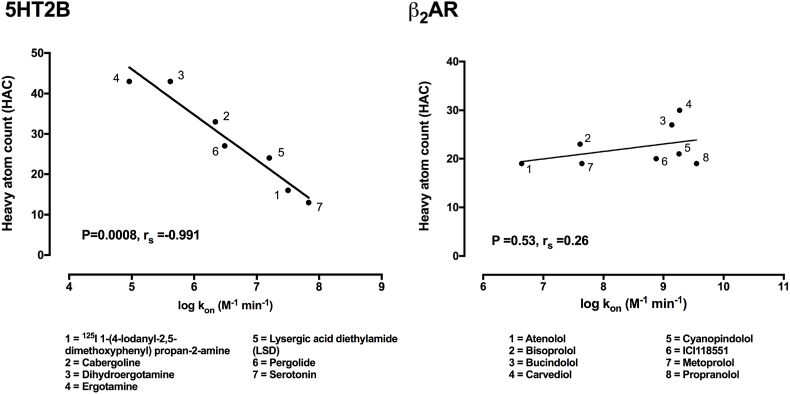
**Correlating 5HT2B and β**_**2**_**AR ligand heavy atom count (HAC) with the measured rate of association (*k***_**on**_**).** Kinetic data are presented as mean values. All 5HT2B kinetic data used in these plots are detailed in Unett et al. (2013) and Wacker et al. (2017). β_2_AR ligand kinetic data used in these plots are detailed in Sykes et al. (2014) Physicochemical properties data were obtained from the National Center for Biotechnology Information. PubChem Compound Database; https://pubchem.ncbi.nlm.nih.gov/compound/. Data was analysed using linear regression in GraphPad Prism v.7 and the relationship between two variables was assessed using a two-tailed Spearman's rank correlation allowing the calculation of the correlation coefficient, r_s_. A P value of 0.05 was used as the cutoff for statistical significance and relationships depicted as trend lines.

In theory drugs with higher molecular mass should show reduced rates of association due to limited access to the binding pocket through what is effectively a narrow passageway (Pan et al., 2013). We have taken data from published studies describing the on-rates of antagonists for the serotonin receptor 2B (5HT2B) and investigated if there is any correlation with heavy atom count (HAC) (Fig. 7; Unett et al., 2013; Wacker et al., 2017). In this example we see how increasing 5HT2B ligand HAC results in a gradual reduction in the measured on-rates of these compounds. Specifically why this should be the case is open to speculation but it is accepted that the 5HT2B receptor possess a ‘lid’ formed by extracellular loop 2 (EL2) residues 207–214 at the entrance to the binding pocket (Wacker et al., 2017). This ‘lid’ effectively covers the binding pocket and may severely restrict the entry and exit of larger molecules. In contrast β_2_AR ligand HAC was not correlated with changes in association rate emphasising the seemingly receptor specific effect of HAC observed with ligands at the 5HT2B receptor. The determinants and importance of association rate are only starting to be uncovered and recognising that both *k*_on_ and *k*_off_ kinetic parameters play a role will lead to a greater understanding of *in vivo* drug action.

In addition, the majority of the studies discussed in this section use synthetic labelled and unlabelled compounds. Endogenous ligands for family A GPCRs of the amine variety tend to show fast on and off-rates (Sykes et al., 2009; Sykes et al., 2012; Klein-Herenbrink et al., 2016) which has made them particularly challenging to measure under physiological conditions. The development of TR-FRET and BRET based methodologies with increased kinetic resolution will aid in the accurate determination of the kinetics of a range of endogenous ligands for family A GPCRs. As concentrations of endogenous ligands can fluctuate widely, especially during synaptic transmission, small changes in occupancy influenced by the *k*_*off*_ of the competing drug, may still allow enough endogenous ligand to bind and elicit a functional response (Vauquelin et al., 2012). Therefore, when determining the influence of binding kinetics on the efficacy of pharmacologically active compounds *in vivo*, it is important to also understand the kinetics of any competing endogenous ligand and the physiological context under which potential competition occurs.

### 5.3 Concluding remarks

In the last ten years there have been huge advances in studying and understanding the importance of binding kinetics at GPCRs. Determining binding kinetics should be central to all drug discovery efforts and with the ever increasing number of GPCR structures and kinetic data sets it should be possible to gain a fuller picture of the molecular determinants of association and dissociation rates. This effort into understanding binding kinetics will in turn help to inform the latest pharmacodynamic models which can be used to predict therapeutic drug actions *in vivo* based partly on their *in vitro* kinetic profiles (Boger et al., 2016; Mager and Krzyzanski 2005; Vlot et al., 2017). Such knowledge has the potential to enhance drug discovery processes and ultimately give rise to new therapeutics with improved properties.
